# French-speaking Swiss physician’s perceptions and perspectives regarding their competencies and training need in leadership and management: a mixed-methods study

**DOI:** 10.1186/s12913-023-10081-x

**Published:** 2023-10-12

**Authors:** R. Lüchinger, M-C. Audétat, N. M. Bajwa, A-C. Bréchet-Bachmann, I. Guessous, H. Richard-Lepouriel, M. Dominicé Dao, Junod Perron

**Affiliations:** 1 Unit of Research and Development in Medical Education, Faculty of Medicine, University of Geneva, Geneva, Switzerland; 2 University Institute of Family and Child Medicine, Faculty of Medicine, University of Geneva, Geneva, Switzerland; 3 Department of Women, Children and Adolescents, University Hospitals of Geneva, Rue Michel-Servet 1, 1211, Geneva, Switzerland; 4 Department of Primary Care Medicine, University Hospitals of Geneva, Geneva, Switzerland; 5 Department of Psychiatry, University Hospitals of Geneva, Geneva, Switzerland; 6 Medical Directorate, University Hospitals of Geneva, Geneva, Switzerland

**Keywords:** Leadership, Management, Competencies, Physicians, Post-graduate training

## Abstract

**Introduction:**

Effective leadership and management (L&M) are essential to the success of health care organizations. Young medical leaders often find themselves ill-prepared to take on these new responsibilities, but rarely attend training in L&M skills. The aims of this study were to evaluate physician’s self-perceived competencies and training needs for L&M, to identify available regional L&M training, and to highlight opportunities, challenges and threats regarding physicians’ training in medical L&M in the French-speaking part of Switzerland.

**Methods:**

We conducted a mixed methods study in three steps: (1) a survey on perceived L&M competencies and training needs (5 dimensions) to all physicians of a Swiss University Hospital (N = 2247); (2) a mapping of the Swiss French speaking L&M training programs through analysis of hospital websites and interviews; and (3) semi-structured interviews with L&M program coordinators about the programs’ strengths and weaknesses as well as the opportunities and challenges to include physicians in such training. We used analysis of variance to compare differences in perceived competences between physicians of different hierarchical status and used Cramer’s V to measure the association’s degree between physicians’ training needs and prior training in L&M and hierarchical status. We analysed semi-structured interviews using thematic analysis.

**Results:**

Five-hundred thirty-two physicians responded (24%). Physicians perceived themselves as rather competent in most leadership dimensions. More experienced physicians reported a higher sense of competence in all dimensions of leadership (e.g. Working with others: *F* = 15.55, *p* < .001; Managing services: *F* = 46.89, *p* < .001). Three competencies did not vary according to the hierarchical status: emotional intelligence (*F* = 1.56, p = .20), time management (*F* = 0.47, p = .70) and communicating (*F* = 1.97, p = .12). There was a weak to moderate association between the responders’ self-perceived needs for training and their hierarchal status for all competencies (Cramer’s V ∈ [0.16;0.35]). Physicians expressed a strong desire to seek out training for all competencies, especially for knowing one’s leadership style (82%), managing teams (83%), and managing conflict (85%). Although existing local L&M training programs covered most relevant topics, only a forth of responders had attended any type of training. L&M program coordinators identified several facilitators and barriers to physician attendance on institutional (matching reality and training), relational (managing collective intelligence), and individual levels (beliefs and self-perceived identity).

**Conclusions:**

French-speaking Swiss hospital physicians clearly express training needs for L&M skills although they only rarely attend such training programs. Reasons for non-attendance to such programs should be explored in order to understand physicians’ low participation rates in these trainings.

**Supplementary Information:**

The online version contains supplementary material available at 10.1186/s12913-023-10081-x.

## Introduction

Effective leadership and management (L&M) are essential to the success of health care organizations and are associated with multiple aspects of organizational performance such as team work, high quality of care, and patient safety [1]. Management involves handling complexity while leadership pertains to dealing with change [2]. More specifically, management relates to the ability to set goals and objectives, create work plans, plan the budget, hire qualified people, communicate goals and how to reach them, delegate work appropriately, monitor work performance, and respond to problems in real time [2]. Leadership relates to the ability to develop a vision and communicate this vision to others, to build support for achieving it, and to encourage others through passion and teamwork to become leaders in turn [3, 4]. Both encourage teamwork to produce high-quality care, support communication and collaboration to maximize performance, and to value actions such as choosing direction, delegating responsibility, and encouraging deliberate action [3].

As physicians progress in their career, they are expected to take on new responsibilities and develop new competencies that are not directly related to their technical expertise. Indeed, patient safety, quality of care, and cost-effectiveness depend not only on physicians’ abilities to decide what care to provide but also on their ability to manage the delivery of these services [5]. High quality care depends on interdisciplinary and interprofessional teamwork. In addition, optimizing team performance requires leadership [6–8]. However, most current leaders in medicine are identified and promoted to leadership positions based on their clinical expertise and their academic performance rather than on the basis of their leadership or managerial skills and experience [9, 10]. Young medical leaders often find themselves ill-prepared to take on these new responsibilities and are frustrated by the lack of training in L&M skills during their undergraduate and postgraduate training [11–15]. Learning on the job and not having the skills to take on their new role can lead to a lack of confidence and poor management performance [9, 16].

As a response to this growing need, various national medical education associations have incorporated L&M dimensions into their conceptual framework and have validated that L&M management competencies should be taught to physicians [17–19]. Several training programs have been developed to improve physicians’ L&M skills in undergraduate, graduate, and continuous medical education [20–24]. Giving the increasing awareness of the importance of L&M skills in Swiss health organisations and medical associations in recent years, numerous initiatives and training courses related to the development of L&M skills for physicians have emerged [25–27].

However, despite the development of national recommendations and training programs in L&M, physician attendance to such programs remains low [28, 29]. In order to understand the mismatch between increasing needs for effective L&M skills among physicians and their low attendance to training programs in L&M, we conducted a study in a Swiss tertiary hospital with the following aims:

- To evaluate physicians’ self-perceived competencies and training needs in a given cultural context.
- To evaluate heads of divisions and department perceptions regarding training needs in L&M skills of their residents, senior residents and attendings.
- To identify training opportunities in medical L&M in the French speaking part of Switzerland.
- To highlight opportunities, challenges and threats regarding physicians’ training in medical L&M.

## Method

### Design and setting

We conducted a mixed-method study including a survey, analysis of the content of L&M training programs and semi-structured interviews between October 2020 and April 2021.

### Online survey on physicians’ perceived competencies and training needs in L&M training

#### Participants

All physicians working at the Geneva University Hospitals were eligible for this study (N = 2247) and were invited to answer an online survey about their perceived competencies and training needs regarding L&M.

The study was granted an exemption by the Ethical Committee for Research of the Canton of Geneva, as it did not involve the collection of any personal health information [30]. The participants were formally informed about the goal of the study and gave written informed consent before answering the survey.

### Questionnaire

The online questionnaire was developed by the whole research team (trained organisational psychologists: RL and MCA; MD and medical educators: NMB, ACB, IG, HRL, NJP) on the basis of several systematic reviews [20, 21, 23, 28] and on the Medical Leadership Competency Framework developed by the Academy of Medical Royal Colleges and the NHS Institute for Innovation and Improvement [18]. This conceptual framework describes the leadership competencies that physicians need in order to become more actively engaged in the planning, delivery and transformation of health care services. This framework includes five dimensions: personal qualities, working with others, service management, service improvement, and setting directions. Items derived from the literature review were categorized according to this model. Two members of the research team, trained in organisational psychology (RL and MCA) checked the meaningfulness of the selected dimensions and items. The questionnaire was pilot-tested by 10 physicians and questions were modified to improve clarity. The final survey included 19 questions on physicians’ self-perceived competencies (Table 1), 19 questions on physicians’ need for training (Table 2) and 19 questions on heads of service’ perceptions of their subordinate training needs (Table 3), using a Likert scale (1 to 4, 1 = poor and 4 = excellent). There were also questions about prior training in management or leadership and sociodemographic characteristics such as age, gender, hierarchy status, medical discipline, and career plan (see Additional file 1 for the full online survey). The questionnaire was sent electronically in October 2020, followed by a reminder 2 and 4 weeks later, using Qualtrics™.

**Table 1:** Physicians’ self-perceived competence regarding management and leadership (Likert scale 1–4, 1 = poor and 4 = excellent)

NHS category	Item	Residents	Chief residents	Attending physicians	Head of department	Differences between groups^a^	
		*M (SD)*	*M (SD)*	*M (SD)*	*M (SD)*	*F*(df1,df2)	*p-value*
Personal qualities	Knowing one’s leadership	2.37 (0.91)	2.46 (0.87)	3.01 (0.75)	3.40 (0.75)	*F*(3,443) = 20.17	*0.001*
	Developing and using one’s emotional intelligence	3.24 (0.74)	3.20 (0.76)	3.33 (0.70)	3.50 (0.69)	*F*(3,481) = 1.56	*0.197*
	Managing one’s time	3.12 (0.70)	3.03 (0.73)	3.11 (0.80)	3.05 (0.60)	*F*(3,488) = 0.47	*0.702*
	Developing professionally	2.90 (0.74)	2.79 (0.68)	3.01 (0.74)	3.45 (0.69)	*F*(3,480) = 5.87	*0.001*
	Acting with integrity	3.56 (0.58)	3.60 (0.58)	3.72 (0.50)	3.85 (0.37)	*F*(3,482) = 3.34	*0.019*
	Being a role model	2.74 (0.77)	2.96 (0.64)	3.43 (0.54)	3.58 (0.51)	*F*(3,426) = 29.19	*0.001*
	Mean Personal qualities	3.00 (0.46)	3.01 (0.44)	3.26 (0.44)	3.47 (0.32)	*F*(3,490) = 15.45	*0.001*
Working with others	Managing a team	2.67 (0.79)	2.97 (0.70)	3.32 (0.59)	3.79 (0.42)	*F*(3,457) = 29.53	*0.001*
	Being involved with the members of one’s team	2.80 (0.77)	3.07 (0.71)	3.18 (0.63)	3.45 (0.60)	*F*(3,457) = 9.81	*0.001*
	Building and maintaining a team spirit	3.00 (0.78)	3.14 (0.70)	3.33 (0.63)	3.68 (0.48)	*F*(3,460) = 8.82	*0.001*
	Managing conflicts	2.73 (0.73)	2.81 (0.67)	2.99 (0.70)	3.45 (0.51)	*F*(3,466) = 8.63	*0.001*
	Communicating internally and externally	3.08 (0.72)	2.96 (0.76)	3.17 (0.77)	3.15 (0.58)	*F*(3,467) = 1.97	*0.118*
	Giving and receiving feedback	2.93 (0.70)	3.03 (0.60)	3.18 (0.63)	3.25 (0.64)	*F*(3,481) = 4.38	*0.005*
	Mean Working with others	2.88 (0.52)	2.99 (0.49)	3.19 (0.47)	3.45 (0.34)	*F*(3,491) = 15.55	*0.001*
Managing services	Managing a project	2.61 (0.83)	2.73 (0.78)	3.20 (0.71)	3.55 (0.51)	*F*(3,442) = 20.61	*0.001*
	Managing resources	1.96 (1.01)	2.02 (0.91)	2.72 (0.96)	3.45 (0.69)	*F*(3,401) = 26.59	*0.001*
	Managing people	1.71 (0.85)	2.06 (0.95)	3.02 (0.72)	3.40 (0.50)	*F*(3,407) = 68.76	*0.001*
	Manage the performance of one’s team	1.77 (0.83)	2.18 (0.79)	2.78 (0.80)	3.35 (0.74)	*F*(3,415) = 47.41	*0.001*
	Mean Managing services	2.11 (0.82)	2.30 (0.69)	2.94 (0.65)	3.43 (0.31)	*F*(3,465) = 46.89	*0.001*
Improving services	Managing change	2.07 (0.90)	2.43 (0.78)	2.98 (0.74)	3.40 (0.50)	*F*(3,418) = 37.30	*0.001*
	Improving the quality / safety of care	2.06 (0.92)	2.53 (0.82)	3.12 (0.68)	3.39 (0.50)	*F*(3,415) = 43.44	*0.001*
	Mean Improving services	2.09 (0.87)	2.50 (0.73)	3.05 (0.63)	3.42 (0.43)	*F*(3,439) = 47.87	*0.001*
Setting direction	Developing a strategic vision and long-term goals	2.03 (0.90)	2.31 (0.83)	2.94 (0.77)	3.55 (0.60)	*F*(3,429) = 40.58	*0.001*

*Note*: *n* residents = 182; *n* chief residents = 144; *n* attending physicians = 126; *n* head of department = 20. a: ANOVA.

**Table 2:** Association between physicians’ self-perceived training needs and absence / presence of prior training in Leader & Management competencies

		Want to attend a training		
NHS category	Items	Trained	Not Trained	Cramer’s V^a^
		*n (%)*	*n (%)*	
Personal qualieits	Knowing one’s leadership	72 (65%)	317 (88%)	0.25**
	Developing and using one’s emotional intelligence	85 (63%)	255 (76%)	0.13*
	Managing one’s time	77 (58%)	250 (74%)	0.15**
	Developing professionally	65 (72%)	299 (78%)	0.06
	Acting with integrity	89 (50%)	177 (60%)	0.10*
	Being a role model	57 (56%)	244 (66%)	0.08
Working with others	Managing a team	75 (66%)	318 (89%)	0.25**
	Being involved with the members of one’s team	77 (66%)	289 (81%)	0.15**
	Building and maintaining a team spirit	72 (69%)	296 (80%)	0.11
	Managing conflicts	98 (77%)	304 (88%)	0.15**
	Communicating internally and externally	69 (64%)	248 (68%)	0.03
	Giving and receiving feedback	118 (58%)	216 (80%)	0.23**
Managing services	Managing project	74 (68%)	289 (80%)	0.12*
	Managing resources	44 (67%)	303 (75%)	0.06
	Managing people	48 (62%)	305 (77%)	0.14*
	Manage the performance of one’s team	44 (64%)	318 (79%)	0.13*
Improving services	Managing change	49 (62%)	318 (79%)	0.17**
	Improving the quality / safety of care	63 (60%)	274 (75%)	0.13*
Setting direction	Developing a strategic vision and long-term goals	47 (66%)	316 (79%)	0.11*

*Note*: *N* = 472; * *p* < .05; ** *p* < .001

a. A value of Cramer’s V within the range of 0.07–0.21 indicates a small effect, a value within the range of 0.21–0.35 indicates a medium effect, and a value larger than 0.35 indicates a large effect

**Table 3:** Heads of department’s perceptions (n = 23) regarding training needs of their residents, chief residents and attendings

NHS category	Item	Residents	Chief residents	Attending physicians
		*n (%)*	*n (%)*	*n (%)*
Personal qualities	Knowing one’s leadership	4 (17%)	8 (35%)	18 (78%)
	Developing and using one’s emotional intelligence	12 (52%)	17 (74%)	17 (74%)
	Managing one’s time	18 (78%)	16 (70%)	13 (57%)
	Developing professionally	13 (57%)	18 (78%)	14 (61%)
	Acting with integrity	16 (70%)	15 (65%)	18 (78%)
	Being a role model	9 (39%)	17 (74%)	17 (74%)
Working with others	Managing a team	4 (17%)	15 (65%)	16 (70%)
	Being involved with the members of one’s team	8 (35%)	11 (48%)	17 (74%)
	Building and maintaining a team spirit	8 (35%)	13 (57%)	17 (74%)
	Managing conflicts	9 (39%)	14 (61%)	18 (78%)
	Communicating internally and externally	5 (22%)	7 (30%)	18 (78%)
	Giving and receiving feedback	9 (39%)	16 (70%)	16 (70%)
Managing services	Managing a project	5 (22%)	11 (48%)	17 (74%)
	Managing resources	4 (17%)	5 (22%)	16 (70%)
	Managing people	3 (13%)	7 (30%)	17 (74%)
	Manage the performance of one’s team	3 (13%)	6 (26%)	17 (74%)
Improving services	Managing change	5 (22%)	7 (30%)	17 (74%)
	Improving the quality / safety of care	11 (48%)	14 (61%)	17 (74%)
Setting direction	Developing a strategic vision and long-term goals	3 (13%)	5 (22%)	16 (70%)

Note: Percentages refer to the Heads of Service’s percentage agreeing on training needs for their residents, chief residents and attendings.

### Mapping of the Swiss French speaking training programs in L&M

One of us (RL) mapped all the L&M training programs offered to physicians in 2021 in the French speaking part of Switzerland using information present on hospital websites and interviews with coordinators of such programs. Information was then categorized and summarized according to the following elements [20, 21, 23, 28]: targeted audience, number of participants, training types, duration and goals, teaching methods, and evaluation types. Any necessary clarification or additional elements were obtained orally with the program coordinators, before finalizing the table (Table 4).

**Table 4:** Summary of the different aspects of the training programs

	Participants	Number participants	Types of training	Training duration	Training goals	Teaching methods	Themes (NHS)	Program evaluation
Training program 1	Team leaders, all health professionals	Min 8 – max 30	« Micro MBA* », CAS** Occasional trainings	3–20 days	Acquire knowledge, Work with partners, Develop skills, Develop awareness of a theme	Role playing, group exercises, reflective work, coaching, theoretical courses	Personal qualities; Working with others; Managing services; Improving services; Setting direction	Participants’ satisfaction
Training program 2	Team leader, unit / department manager	Min 15	CAS	315 h	Manage projects, Develop transferable knowledge in different contexts	Course + personal work	Working with others; Managing services;	
Training program 3	Managers or future managers in health network		CAS	15–21 days	Develop expertise, Manage change, Use a methodological approach Develop leadership skills	Knowledge and reflective practice, personal work	Personal qualities; Managing services; Improving services;	
Training program 4	New or experienced managers	Max 30	CAS / MAS***, occasional trainings, Introduction	2 h – 6 semesters	Develop managerial skills Work on a project with follow-up Training for new functions	e-learning, interactive teaching, coaching, face-to-face and distance learning	Personal qualities; Working with others; Managing services; Improving services; Setting direction	
Training program 5	Middle managers or institutional leaders			10.5 – 600 h	Acquire skills for a position Prepare for federal exams; Acquire new concepts; Implement continuous improvement in context (environment)	Interactive, role-playing, theoretical contributions, practical application, work in sub-groups	Personal qualities; Working with others; Managing services; Improving services	Participants’ satisfaction
Training program 6	Hospital managers, health, administrative or insurance staff		CAS / MAS	5 months – 3 years	Acquire skills in health organization		Managing services; Improving services; Setting direction	

*Note*: *MBA: Master in Business Administration; **CAS: Certificate of Advanced Studies; ***MAS: Master of Advanced Studies

#### Semi-structured interviews with coordinators of L&M training programs

Finally, RL contacted via email and phone all coordinators of the L&M training programs existing for physicians in the French speaking part of Switzerland (n = 5) and a key teacher’s (involved in several programs) (N = 6) to explore their perceptions and perspectives through semi-structured interviews. Four agreed to be interviewed and signed the consent form (see Additional file 3). The interview guide included additional questions about teachers’ and coordinators’ roles in leadership or management trainings, the characteristics of the trainings such as the targeted audience, the teaching methods, the taught competencies, and finally the strengths and weaknesses of training programs. They were also asked through open-ended questions about the opportunities, challenges, and threats regarding physicians’ attendance to such programs (see Additional file 2 for the interview guide). RL conducted the interviews. They lasted between 43 min and 1 h and 13 min and were audio recorded and transcribed verbatim.

### Analysis

#### Survey

Data from the survey were analysed descriptively using means and percentages. After checking assumptions for normality, homoscedasticity and multicollinearity, we used analysis of variance to compare differences in perceived competencies between physicians of different hierarchical status and Post-Hoc multiple comparisons were conducted using Tukey’s range test. We used Cramer’s V to measure the association between physicians’ prior trainings in L&M (attendance / no attendance) and physicians’ needs for trainings in L&M (willing to / not willing to attend trainings) for each NHS categories’ items. Cramer’s V is a normalized version of the *χ*2 test statistic. It is used to compare the strength of the relationship between the two nominal variables under study [31]. A value of Cramer’s V within the range of 0.07–0.21 indicates a small effect, a value within the range of 0.21–0.35 indicates a medium effect, and a value larger than 0.35 indicates a large effect (i.e. a large effect indicates a high association between the absence of prior training and the will to attend training in L&M). All analyses were run on IBM SPSS Statistics v.25. Free text fields for open-ended questions regarding prior training in L&M were analysed thematically and categorized by RL and NJP into Geneva/Swiss/abroad medical leadership oriented trainings and other kinds of training unrelated to health management/leadership.

### Semi-structured interviews

We followed Standards for Reporting Qualitative Research (SRQR) [32] and performed a thematic analysis of the semi-structured interviews based on a descriptive phenomenological approach [33]. This approach allowed us flexibility to examine the coordinator’s experience in an explicit way, maintaining methodological consistency across all interviewees [34]. After reading and discussing the content of five transcripts, two researchers (RL and NJP) conducted a SWOT analysis (strengths, weaknesses, opportunities and threats): - strengths and weaknesses of the Swiss French speaking training programs in L&M - opportunities and threats regarding physicians‘ participation to such programs [35]. NJP coded all the transcripts and RL checked the coding. Throughout the analytic process, any divergences between them were discussed until consensus was reached.

## Results

### Participants’ socio-demographics and training experience in L&M

Out of all contacted physicians, 532 (24%) responded to the study. About less than a third of physicians across the different hierarchical levels participated to the study with 23% of the Residents, 23% of the Chief residents, 26% of the Attending physicians and 34% of the Heads of department. As shown in Table 5, main physicians’ medical specialities were internal medicine, psychiatry, paediatric, emergency medicine and surgery. For two third of them, their career plan at 5 years was to work within the institution, but this percentage decreased to less than half at 10 years. The higher the hierarchical level was, the more participants attended training related to L&M. One hundred thirty-seven (25%) participants reported having attended one or more trainings in L&M Participants mentioned 157 different types of training. They included courses within the Geneva University Hospitals (n = 58, 37%), courses in other Swiss institutions or organisations (n = 50, 32%), courses abroad or in foreign institutions (n = 17, 11%) and other kinds of training such as coaching, military training or non-specified (n = 32, 20%).

**Table 5:** Socio-demographics

**Gender**	n		*%*	
Female	275		52%	
**Hierarchical level**	n		%	
Residents	209		39%	
Chief residents	158		30%	
Attending physicians	142		27%	
Heads of department	23		4%	
**Speciality**	n		%	
Internal medicine	179		34%	
Psychiatry	93		17%	
Pediatric	59		11%	
Emergency medicine	54		10%	
Surgery	52		10%	
Gyneco-obstetrics	18		3%	
Neurology	15		3%	
Radiology	9		2%	
ORL	8		2%	
Hematology	7		1%	
Others	38		7%	
**Career plan**	at 5-years		at 10-years	
	n	%	n	%
Work in institution	390	73%	244	46%
Private practice / centres	49	9%	136	26%
Other	30	6%	50	9%
Undecided	63	12%	102	19%
**Attented training(s) in L&M**: n = 137 (25%)	n		%	
Residents	20		10%	
Chief residents	33		21%	
Attending physicians	68		48%	
Heads of department	16		70%	

### Participants’ self-perceived competencies in L&M

The participants perceived themselves as rather competent in most leadership dimensions (Table 2). They reported stronger self-perceived competencies in *personal qualities*, *working with others*, and *managing services* dimensions than *improving services* and *setting direction* dimensions. As participants had a higher hierarchical status, they tended to perceive themselves as more competent in most dimensions (Table 2). Residents’ lowest self-perceived competencies were related to *managing people* and endorsing high responsibility inside the service (e.g. *personnel or resource management*, *improving quality* or *setting strategic goals*). All the participants indicated a high level of competence regarding *behaving with integrity*, independent of their hierarchal status. Self-perceived competencies such as *developing and using one’s emotional intelligence* (*F*(3,481) = 1.56, *p* = .20), time management (*F*(3,488) = 0.47, *p* = .70) and *communicating internally and externally* (*F*(3,467) = 1.97, *p* = .12) did not change according to the hierarchical status. After adjustment for hierarchical status, we found no gender differences regarding self-perceived competence.

### Participants’ self- perceived training needs in L&M

Most participants expressed training needs in L&M, especially for competencies such as *knowing one’s leadership style*, *managing a team*, and *managing conflicts* (Table 3). Having attended a prior training in L&M was only slightly associated with lower perceived needs of further training (Cramer’s V∈ ]0.11;0.25]) for most competencies (Table 3). However, there was no such association between training (or not) and perceived training needs for competencies such as developing professionally, *being a role model*, *communicating internally and externally*, and *managing resources*.

There was a weak to moderate association between the responders’ self-perceived needs for training and their hierarchal status for all competencies (Cramer’s V ∈ [0.16;0.35]) excepted for *communicating internally and externally* that showed no association. In other words, as participants’ hierarchical level increased, their perceived need for training slightly or moderately decreased.

### Heads of department’s perceptions regarding staff training needs in L&M

The heads of department perceived different training needs for the physicians of their teams (Table 4). They mainly promoted training in the *personal qualities* dimension for residents while they favoured training in *personal qualities* and *working with others* dimensions for chief residents. They considered that the L&M training should cover all the competencies of the NHS conceptual framework used for attending physicians.

### Local training programs in management and leadership

We identified six training programs in the French speaking part of Switzerland. From the six identified hospital programs, four coordinators agreed to participate in the study and completed the information extracted from the websites of the training programs in medical L&M. Two of the hospitals offering training programs accounted for more than 10’000 collaborators (5’000 and 2’000 collaborators respectively). The characteristics of the training programs are summarized in Table 5. L&M training programs varied in terms of goals and duration according to the hierarchical level and training needs of the target audience and the size of the institution. The target audience was multidisciplinary and interprofessional. The training dimensions selected varied according to the type/duration of different trainings. Four hospital training centers addressed *Personal Qualities* competencies (87.5%), *Working with other* competencies (100%), 87.5% of *Managing services* competencies (87.5%), *Improving services* competencies (88%), and *Setting direction* competencies (75%) in their programs. The impact of the training effectiveness was either not assessed or based on participants’ satisfaction (level 1 of Kirkpatrick’s model of training evaluation [36]).

Four coordinators and one facilitator involved in two different training programs were interviewed. Their perceptions regarding strengths and weaknesses of their L&M trainings as well as opportunities and challenges of physicians’ participation in such programs are summarized below (for illustrative quotes, see Table 6. MDD and NMB, English native speakers, translated the transcript from French to English. They took care to ensure high-quality translation for all qualitative data.

**Table 6:** Coordinators and trainer’s quotes about opportunities, challenges and threats regarding physicians’ training in leadership & management

	Themes	Quotes
Opportunities	1. Cultural change of leadership/management among institutions	- *So, by themselves, by the regulations, and then we also have senior doctors who are promoters, well, there you go, who see the benefit of training their young managers. They also delegate more managerial activities, so we also have quite a few promoters in the heads of service, heads of department, so we are lucky. Coordinator Nr. 2* - *It has become, punctually, because the new generation of doctors is different from the previous one. The previous one, in fact, there was no need for leadership, they had it naturally, all these skills. And then, well I see that with the new generation, I … our medical director who came to us in 2013, that maybe. Uh, as soon as he arrived we promoted him to medical director because the old one quit. And then he already came with a CAS in management, and then he was always very interested in that, and then now, we are starting to talk about it, then to think about what we could put in place. Coordinator Nr 4*
	2. Integration of L&M training into physicians’ institutional career development plan	- *So, the strengths are clearly that they understand you quickly, they often have a very strong interest in these themes, more and more, in fact. At the beginning we had the impression that we had to go and get them, as I said, it’s compulsory, but more and more people are saying to us, well, I’m going to be promoted or I have a career plan, I’d like to sign up now. So, in fact they are voluntary, they are more tagged, they are voluntary, they understand quickly, that they have an interest in so that’s the strengths. Coordinator Nr. 2*
	3. Physicians’ prior training/experience with communication and pedagogical skills	- *Yes, there are many who, for example, have learned how to break bad news to a patient, how to manage a conflict between a family and a caregiver, and how to conduct annual evaluation interviews. There are many who have learned this and then mastered it. And who just didn’t understand that they could transfer the same learning into the management of the staff. Trainer Nr 1* - *uh as I said, there’s this parallel with the biology and medical school, where we’ve worked with them quite a bit. Then, they see more and more, uh how they do pedagogy or support, uh typically in relation to young assistant doctors on feedback etc., then they can make the parallel on the management of their own team. Coordinator Nr. 2*
Challenges/threats	1. Physicians’ high expectations regarding the cost-effectiveness of the training	- *I think clearly, we need to balance the cost-benefit, that is to say the cost of my training and the benefit I will get from it, I think… in any case, with the doctors it is key… Coordinator Nr. 2* - *Yes, so clearly to see, uh to see very quickly that is to say that the training must go quickly too, must often it is nevertheless, of it is necessary an individual benefit, the collective benefit it interests a little less… So it is necessary that the benefit is directly exploitable of return on the ground. Coordinator Nr. 2*
	2. Mismatch between training and the reality of work	- *I often hear that there are differences between what we teach in management because they see it in practice. We teach benevolence in management, it’s the benevolent management set, and then I often have people tell me that it’s especially at the level of doctors “but Marie-Pierre, you don’t know how we’re spoken to in the operating room on the teacher, well when he’s not happy we try to negotiate with him, he doesn’t talk to us anymore, he sends us on our way”. Ah well, there is a difference between what we teach and the culture in which they live. Trainer Nr 1* - *But that’s one of the difficulties in training. It’s that often the models are contradictory to what we teach them. Trainer Nr 1*
	3. Physicians’ discomfort in collaborating with others	- *Because, what is very complicated, and I think, that somewhere doctors, have the posture of leader rather quickly. That is to say, I will specify what I mean by the posture of leader, but they develop quite easily and for quite a long time strategic visions. It’s not so much on that that complexities can be found, what is more complicated for them is the managerial aspect, i.e. how to manage. And then, I think that where we also have a big change to make is on the integration of stakeholders, that is to say how to develop collective intelligence …. The question today is not that, it’s from that, from your visions, how do you create change? How do you get your teams to make changes? Coordinator Nr 3*
	4. Physicians’ personal beliefs	- *Often they will change their mind after the first module, they will be quite happy. But there may always be one doctor who doesn’t understand. Not that we train them in management, but they don’t even understand that we ask them to do management. And they say to themselves but I’m a doctor, I studied to be a doctor, I didn’t study to give feedback to people and to lead meetings. Trainer Nr 1*
	5. Physicians’ reluctance to reveal their weaknesses	- *The second difficulty, for me, is the difficulty that some people have in putting themselves into play. And when I say to put oneself in play, it is in the two senses of the term play, it is the personal implication, it is not to be afraid to show its weaknesses, to say its difficulties, that for several it is complicated. And there is also the game. That is to say, as soon as you want to get into role-playing or simulation models, it is quickly complicated. And this is found in particular in the medical profile, but also in others. But in any case, there are strong underlying representations and this difficulty of getting into training. We’re still dealing with a question of posture that we mentioned before. Coordinator Nr 3*

### Strengths and weaknesses of training programs

Most coordinators considered that training programs had to adapt to participants’ needs and/or articulate with personal and institutional needs to be successful. This was especially the case in one hospital where leadership training programs were closely linked to new values the institution wanted to promote. The fact that training programs commonly involved health professionals of different backgrounds, included multiple teaching formats, and ensured group continuity were reported as strengths. Follow-up in the workplace was considered as crucial for the success of training and transfer.

The main difficulties they encountered were participants with different needs, insufficient interactive teaching methods, and a lack of coaching of workplace-based projects in the post-training phase.

Coordinators and trainers perceived several opportunities and challenges/threats regarding physicians’ training in L&M. They are mentioned in Table 6 (themes and quotes).

### Opportunities regarding physicians’ training in leadership & management

The main opportunities to further develop a culture of leadership/management among physicians related to current cultural institutional changes with heads of service or medical directors promoting more informed leadership and supporting L&M training.

Coordinators/trainers acknowledged that physician participants were motivated and interested, especially when a training in L&M was part of their institutional career development plan. They perceived that the fact that some institutions made the training compulsory for physicians taking institutional responsibilities was a strong incentive and did not negatively affect physician’s attitudes toward L&M.

They considered that physicians usually mastered communication skills useful for difficult situations because they practised these skills in their daily clinical work but were unaware that they could transfer these skills to other situations such as team management.

The similarity and synergy between skills and attitudes acquired in both pedagogical and L&M trainings were seen as beneficial and mutually reinforcing.

### Challenges/threats regarding physicians’ training in L&M

Participants identified challenges and threats at different levels. On the institutional level, they reported that physicians more than other health professionals, had little protected time to dedicate to such training and expected high cost-effectiveness and quick individual benefits. A mismatch between what was taught and what really happened in the workplace in terms of L&M was seen as a threat to skill transfer.

When relating to the physician’s position inside a group, some coordinators highlighted the imbalance between physicians’ comfort in endorsing a position of leader and developing a strategic vision, their difficulty in managing teams, and in managing collective intelligence.

Finally, on the individual level, they reported that some physicians were reluctant to develop leadership & management skills because they believed that being a manager was not part of their professional identity. Some coordinators also felt that some physicians were disinclined to practice role-playing during training by fear of revealing their lack of competence or vulnerability. They considered such an attitude to be a threat to training.

## Discussion

The aim of this study was to evaluate Swiss physicians’ self-perceived competence and training needs in L&M, to map the local training offers in L&M, and to explore opportunities, challenges, and threats regarding physician’s training in such a field. We showed that self-perceived competence grows and training needs decrease as physicians attain a higher hierarchical status. However, self-perceptions regarding competencies such as using one’s emotional intelligence, communicating, and managing one’s time did not change according to physicians’ hierarchical status. Physicians expressed high training needs for all competencies, especially for knowing one’s leadership, managing teams, and managing conflict. Actual local training programs in L&M cover a large spectrum of relevant competencies that could meet participants’ needs and expectations. However, only a fourth of participants attended any type of training in L&M. In order to make such training programs attractive for physicians, program coordinators highlighted several factors of success: to value leadership skills and culture, to reinforce positive leadership role modelling on the institutional level, and to support and integrate more explicitly leadership training into physicians’ institutional career development plan.

Perceived self-competence in L&M increase and training needs decreased as physicians have higher hierarchical status. The fact that work experience plays an important role in the development of self-confidence in L&M managerial skills is not surprising since physicians mainly learn such skills on the job. Since training needs and skill growth were self-perceived and were not verified, the perception of decreasing training needs could also be attributed to overconfidence. However, our results are in line with Lau et al. who reported similar observations among nurses [37].

We showed that self-perceived skills regarding emotional intelligence, communication, and time management did not tend to improve as physician have more professional experience and endorse more responsibilities. Emotional intelligence, defined as the ability to perceive and express emotion, assimilate emotion in thought, understand and reason with emotion, and regulate emotion in the self and others is considered a key element of leadership that can be taught and assessed [38]. Lack of self-perceived improvement in emotional intelligence can be explained by several reasons: physicians may have not attended any training in this field or the training that they attended did not address this dimension. Indeed, most training programs mostly focus more on “know” and “do” elements rather than on “be” elements such as self-awareness and emotional intelligence [20, 39]; improving emotional intelligence through training might be challenging as the impact of training on physicians’ emotional intelligence remains unclear [38]. Finally, physicians may believe that similar to patient-physician communication, emotional intelligence is intrinsic and cannot be taught. The fact that time management remains a challenge for all physicians at all levels of professional experience is not surprising since non clinical tasks, new roles and responsibilities, and involvement in various management projects increase and add to clinical activities as physicians move to higher leadership positions [40].

Healthcare professionals usually express strong interest and training needs for L&M regardless of their level of training [29, 41]. This may indicate a cultural or generational shift among physicians since previous studies showed their lack of interest in management and leadership skills [16, 42]. However, this need for training and skill growth was examined as a self-perceived need and therefore was not verified. The perception of decreasing training needs could also be attributed to overconfidence. Similarly to our study, interest for L&M skills related to team leading, resolving conflicts, and self-awareness has been reported as particularly high [28, 43, 44] and are in line with reports of clinical leadership effectiveness [5, 45]. However, despite interest and availability of existing local L&M training programs covering such topics, only a few physicians take advantage of these learning opportunities in Switzerland [29]. Reasons for non-attendance may include a lack of time, competing priorities, crowded pre- and post-graduate curricula, and a lack of institutional support [42, 46–49]. Residents spend their time acquiring clinical skills and their exposure to leadership skills generally relies upon role models that have not been themselves trained in such skills and learn and apply through trial and error [43]. Physicians, known to be individualist and value autonomy, may be reluctant to acknowledge a need for training [5] and learn collectively with other health professionals. However, this hypothesis does not seem to be supported by our results, indicating an initial interest for training. Although only a minority had experienced some form of training. Finally, health organisations and training institutions contribute to such phenomenon by moving physicians into leadership roles without requesting appropriate training and skills [5]. Though, there is some evidence that effective physicians’ leadership skills lead to better patient and organisational outcomes [50–53].

### Implications

This study highlights some implications for the teaching and development of L&M skills for physicians. Firstly, these skills must be addressed early in physicians’ education. We have shown that junior physicians are interested in training in L&M skills. Introducing this training to medical students may be even more relevant, as the development of L&M skills may raise young physicians’ awareness of their role as leaders in clinical practice [5, 21, 54–57]. By anticipating their needs, based on the experience of today’s physicians, we can equip future physicians with L&M skills that will be useful for the situations they will encounter later in their practice.

Secondly, clinical teaching and L&M skills share many similarities such as understanding and taking into account others’ perspectives and needs, supporting change and development, being inspirational and a positive role model, or sustaining networks with L&M skills [58–60]. The importance of effective teaching skills is now widely recognized. Most training health institutions provide Faculty training programs [20, 21, 24, 55, 56]. Including L&M skills training into such programs may help physicians integrate and enrich their different professional identities of clinicians, teachers, and leaders in a coherent way, and provide links between quality of care, effective teaching and team management skills.

Thirdly, key factors of success of L&M programs mentioned by the coordinators of Swiss L&M training programs are supported by the literature. Institutional support is critical to the success of such training programs [23] and the implementation of L&M training must always be accompanied by a clear institutional will and strategy [61, 62]. To make training in L&M skills mandatory, to implement a longitudinal curriculum from graduate to continuous training, and to reward effective medical leaders are powerful and complementary ways to change physicians’ mentality and may help them to consider L&M to be part of their professional identity [5, 23, 62, 63]. This means not only considering the appropriateness of institutional goals for the missions entrusted to physicians, but also communicating and understanding these goals.

#### Limitations

There are several limitations to this study. First, the physicians’ response rate was quite low. This may be explained by the format of the survey (online) and the COVID pandemic prevailing during the data collection time. However, the response rate was consistent with the literature [64]. It is possible that we selected only the physicians mostly interested in L&M and their self-perceived competencies and needs were under or over-estimated. However, our results regarding self-perceived competencies’ and needs’ levels are in line with several studies [5, 10, 28, 37, 40, 42–44]. Second, we collected physicians’ self-perceived competence in L&M and it is known that self-perceptions are influenced by desirability bias [65]. Exploring more qualitatively physicians’ perceptions regarding L&M is warranted in order to broaden our understanding on what can be done to improve competence in L&M. Third, we conducted the survey in a single tertiary hospital and mapped the existing L&M training only to the French speaking part of Switzerland. This may affect the generalizability of our findings. With the current trend and the development of new L&M training for healthcare professionals in Switzerland, we encourage replication of the online survey and more complete mapping of the current L&M training in other parts of Switzerland since the culture influences physicians’ needs and priority regarding L&M [29].

## Conclusion

Swiss hospital physicians express real training needs for L&M skills although they perceive themselves to be more competent as they have more leading positions. However, only a minority of them have attended any L&M training programs although local training opportunities exist. Reasons for non-attendance to these programs should be explored more in depth in order to understand physicians’ reluctance to training despite evidence that effective physicians’ leadership skills lead to better patient and organisational outcomes.

### Electronic supplementary material

Below is the link to the electronic supplementary material.

Additional file 1

Additional file 2

Additional file 3

## Data Availability

The datasets used and/or analysed during the current study are available from the corresponding author on reasonable request.
