# Supplementary material for: French-speaking Swiss physician’s perceptions and perspectives regarding their competencies and training need in leadership and management: a mixed-methods study
Source: BMC Health Serv Res. 2023 Oct 12;23:1095. doi: 10.1186/s12913-023-10081-x (PMC10571431; doi:10.1186/s12913-023-10081-x)
Supplement: Supplementary file 1 — Additional file 1 [file 12913_2023_10081_MOESM1_ESM.pdf]

Project on leadership/management training needs
Survey Flow

Block: Introduction (2 Questions)
Standard: Initial Question (1 Question)
Standard: Questionnaire (19 Questions)
Standard: Demographics and End of Questionnaire (6 Questions)

Page Break

Start of Block: Introduction

Q1 Dear colleagues,

You are invited to participate in a survey conducted by the Institute of Primary Care Medicine (IMPR) of the HUG. The purpose of this survey is to evaluate your training needs as an intern, a clinic manager, a physician assistant or a physician manager in the field of leadership/management. Your participation consists of completing a questionnaire that should only take 10-15 minutes.

This survey is supported by the medical management of the HUG.

You are free to accept or refuse to participate in the survey. The data collected for research purposes are anonymous. The analyzed results may be used for scientific publications. The confidentiality of the data will be ensured, and your name or any information that could identify you will not appear anywhere.

Q2 Do you agree to participate in this study?

- Yes (1)
- No (2)

Skip To: End of Survey If Acceptez-vous de participer à cette étude? = Non

End of Block: Introduction

Start of Block: Question initiale

Q3 What is your current hierachical status?

- Resident (1)
- Chief resident (2)
- Attending physician (3)
- Head of department (4)

End of Block: Question initiale

Start of Block: Questionnaire

Q4 What is your 5-year career plan?

- ◯ Institute (1)
- ◯ Private practice in an office/medical center (2)
- ◯ Other (please indicate below) (3) ____
- ◯ I don't know (4)

Q5 What is your 10-year career plan?

- ◯ Institute (1)
- ◯ Private practice in an office/medical center (2)
- ◯ Other (please indicate below) (3) ____
- ◯ I don't know (4)

Q6 What type of leadership/management responsibilities do you currently have? (Please describe)

____

____

____

____

____

Q7 What type of leadership/management responsibilities do you see yourself having in 5 years? (Please describe)

____

____

____

____

____

Q8 Have you ever attended a leadership/management training course?

- ○ Yes (1)
- ○ No (2)

Display This Question:

If Avez-vous déjà suivi une formation en leadership / management? = Oui

Q9 What training have you attended?

- ☐ CAS / DAS / MAS HUG management in health institutions (Unige) (1)
- ☐ Training at the Royal College of Physician in Luzern (ISFM) (2)
- ☐ Introduction to management, medical track HUG (5 modules) (3)
- ☐ Training within your department (4)
- ☐ Other (please specify below) (5)

____

Display This Question:

If Quelle.s formation.s avez-vous suivie.s? = CAS / DAS / MAS HUG management dans les institutions de santé (Unige)

Q10 You have mentioned that you have followed a CAS / DAS / MAS HUG management course in health institutions (Unige), which one(s)?

____

____

____

____

____

Display This Question:

If If Vous avez indiqué avoir suivi une formation CAS / DAS / MAS HUG management dans les institutions... Text Response Is Not Empty

Q11 Was this training (CAS / DAS / MAS HUG) useful to you?

- Yes (1)
- No (2)

Display This Question:

If Cette formation (CAS / DAS / MAS HUG) vous a-t-elle été utile? = Oui

Or Cette formation (CAS / DAS / MAS HUG) vous a-t-elle été utile? = Non

Q12 Why?

____

____

____

____

____

Display This Question:

If Quelle.s formation.s avez-vous suivie.s? = Formation au sein de votre département

Q13 You have mentioned that you have attended a training course in your department, on what topics and for how long?

____

____

____

____

____

Display This Question:

If If Vous avez indiqué avoir suivi une formation au sein de votre département, sur quel-s thème-s et d... Text Response Is Not Empty

Q14 Was this training useful to you?

○ Yes (1)

○ No (2)

Display This Question:

If Cette formation vous a-t-elle été utile? = Oui

Or Cette formation vous a-t-elle été utile? = Non

Q15 What are the reasons for this?

____

____

____

____

____

Display This Question:

If Quelle.s formation.s avez-vous suivie.s? = Formation Royal College of Physician à Lucerne (ISFM)

Q16 You have indicated that you have attended the "Royal College of Physician training in Luzern (ISFM)". Was this training useful to you?

- ◯ Yes (1)
- ◯ No (2)

Display This Question:

If Vous avez indiqué avoir suivi la formation "Formation Royal College of Physician à Lucerne (ISFM)... = Oui

Or Vous avez indiqué avoir suivi la formation "Formation Royal College of Physician à Lucerne (ISFM)... = Non

Q17 What are the reasons for this?

____

____

____

____

____

Display This Question:

If Quelle.s formation.s avez-vous suivie.s? = Initiation au management, filière médicale HUG (5 modules)

Q18 You have mentioned that you have taken the "Introduction to management, HUG medical field (5 modules)" training. Was this training useful to you?

- ◯ Yes (1)
- ◯ No (2)

Display This Question:

If Vous avez indiqué avoir suivi la formation "Initiation au management, filière médicale HUG (5 mod... = Oui

Or Vous avez indiqué avoir suivi la formation "Initiation au management, filière médicale HUG (5 mod... = Non

Q19 What are the reasons for this?

____

____

____

____

____

Page Break

Q20 How would you rate your competence in the following leadership/management areas:

	Poor (1)	Fair (2)	Good (3)	Excellent (4)	I don't know (5)
Knowing one's own leadership (e.g., leadership styles) (1)	○	○	○	○	○
Developing and using emotional intelligence (e.g., perceiving, using, understanding one's own or others' emotions) (2)	○	○	○	○	○
Managing one's time (e.g., setting priorities / preventing burnout) (3)	○	○	○	○	○
Developing professionally (e.g., managing career progression) (4)	○	○	○	○	○
Acting with integrity (e.g., ethically, inclusively and fairly) (5)	○	○	○	○	○
Being a role model (e.g., inspiring others) (6)	○	○	○	○	○
Managing a team (e.g. motivating a team / managing a meeting / etc.)	○	○	○	○	○

(7)					
Being involved with team members (e.g. identifying and preventing burnout / helping with personal development / etc.) (8)	○	○	○	○	○
Building and maintaining team spirit (e.g., ensuring team cohesion) (9)	○	○	○	○	○
Managing conflicts (e.g., conflict of interest or opinion) (10)	○	○	○	○	○
Communicating internally and externally (e.g. intra / extra HUG) (11)	○	○	○	○	○
Giving and receiving feedback (12)	○	○	○	○	○
Managing a project (13)	○	○	○	○	○
Managing resources (e.g., developing, structuring, and managing a budget / allocating resources) (14)	○	○	○	○	○
Managing staff (e.g. selecting, recruiting and hiring staff / conducting	○	○	○	○	○

appraisal interviews / setting goals / delegating tasks) (15)					
Managing team performance (e.g., analyzing and promoting performance) (16)	○	○	○	○	○
Managing change (e.g., implementing and supporting change / stimulating innovation) (17)	○	○	○	○	○
Improving the quality/safety of care (e.g. in a unit or department) (18)	○	○	○	○	○
Developing a strategic vision and long-term goals (19)	○	○	○	○	○

Page Break

Q21 How would you rate your training needs in the following leadership / management areas:

	I have already received training and that is enough for me (1)	I have already received training and would like to receive further training (2)	I have not received any training and wish to attend training (3)	I have not been trained and do not wish to be trained (4)
Knowing one's own leadership (e.g., leadership styles) (1)	○	○	○	○
Developing and using emotional intelligence (e.g., perceiving, using, understanding one's own or others' emotions) (2)	○	○	○	○
Managing one's time (e.g., setting priorities / preventing burnout) (3)	○	○	○	○
Developing professionally (e.g., managing career progression) (4)	○	○	○	○
Acting with integrity (e.g., ethically, inclusively and fairly) (5)	○	○	○	○
Being a role model (e.g., inspiring others) (6)	○	○	○	○
Managing a team (e.g. motivating a team / managing a meeting / etc.) (7)	○	○	○	○

Being involved with team members (e.g. identifying and preventing burnout / helping with personal development / etc.) (8)	○	○	○	○
Building and maintaining team spirit (e.g., ensuring team cohesion) (9)	○	○	○	○
Managing conflicts (e.g., conflict of interest or opinion) (10)	○	○	○	○
Communicating internally and externally (e.g. intra / extra HUG) (11)	○	○	○	○
Giving and receiving feedback (12)	○	○	○	○
Managing a project (13)	○	○	○	○
Managing resources (e.g., developing, structuring, and managing a budget / allocating resources) (14)	○	○	○	○
Managing staff (e.g. selecting, recruiting and hiring staff / conducting appraisal interviews / setting goals /	○	○	○	○

delegating tasks) (15)				
Managing team performance (e.g., analyzing and promoting performance) (16)	○	○	○	○
Managing change (e.g., implementing and supporting change / stimulating innovation) (17)	○	○	○	○
Improving the quality/safety of care (e.g. in a unit or department) (18)	○	○	○	○
Developing a strategic vision and long-term goals (19)	○	○	○	○

Page Break

Display This Question:

If Quel est votre statut? = Chef-fe de service

Q22
You have indicated that you are a head of department. Please indicate the leadership/management issues that you consider to be a priority for your residents, chief residents and attending physicians (multiple choices possible):

	Residents (1)	Chief residents (2)	Attending physicians (3)
Knowing one's own leadership (e.g., leadership styles) (1)	☐	☐	☐
Developing and using emotional intelligence (e.g., perceiving, using, understanding one's own or others' emotions) (2)	☐	☐	☐
Managing one's time (e.g., setting priorities / preventing burnout) (3)	☐	☐	☐
Developing professionally (e.g., managing career progression) (4)	☐	☐	☐
Acting with integrity (e.g., ethically, inclusively and fairly) (5)	☐	☐	☐
Being a role model (e.g., inspiring others) (6)	☐	☐	☐
Managing a team (e.g. motivating a team / managing a meeting / etc.) (7)	☐	☐	☐
Being involved with team members (e.g. identifying and preventing burnout / helping with personal development / etc.) (8)	☐	☐	☐
Building and maintaining team spirit (e.g., ensuring	☐	☐	☐

team cohesion) (9)			
Managing conflicts (e.g., conflict of interest or opinion) (10)	☐	☐	☐
Communicating internally and externally (e.g. intra / extra HUG) (11)	☐	☐	☐
Giving and receiving feedback (12)	☐	☐	☐
Managing a project (13)	☐	☐	☐
Managing resources (e.g., developing, structuring, and managing a budget / allocating resources) (14)	☐	☐	☐
Managing staff (e.g. selecting, recruiting and hiring staff / conducting appraisal interviews / setting goals / delegating tasks) (15)	☐	☐	☐
Managing team performance (e.g., analyzing and promoting performance) (16)	☐	☐	☐
Managing change (e.g., implementing and supporting change / stimulating innovation) (17)	☐	☐	☐
Improving the quality/safety of care (e.g. in a unit or department) (18)	☐	☐	☐
Developing a strategic vision and long-term goals (19)	☐	☐	☐

End of Block: Questionnaire

Start of Block: Données démographiques et Fin du questionnaire

Q23 What year were you born?

____

Q24 What is your gender?

- ○ Male (1)
- ○ Female (2)
- ○ Other (3)

Q25 In what year did you obtain your federal medical degree?

____

Q26 What is your main medical discipline?

- ◯ Internal Medicine (general outpatient, general hospital, geriatrics, rehabilitation, subspecialties) (1)
- ◯ Intensive care medicine (12)
- ◯ Surgery (all specialties) (3)
- ◯ Dermatology (11)
- ◯ Gyneco-obstetrics (4)
- ◯ Neurology (5)
- ◯ ENT (6)
- ◯ Ophtalmology (7)
- ◯ Pediatrics (outpatient, inpatient, subspecialty) (8)
- ◯ Psychiatry and child psychiatry (9)
- ◯ Radiology (13)
- ◯ Other (please specify below) (10) ____

Q27 You have completed this questionnaire. Would you like to be part of a focus group on leadership/management training? If so, please enter your email address so we can contact you.

- ◯ Yes (please indicate below) (1) ____
- ◯ No (2)

Q28 Thank you for participating in this study. You can now close the questionnaire.

End of Block: Données démographiques et Fin du questionnaire
