# Supplementary material for: French-speaking Swiss physician’s perceptions and perspectives regarding their competencies and training need in leadership and management: a mixed-methods study
Source: BMC Health Serv Res. 2023 Oct 12;23:1095. doi: 10.1186/s12913-023-10081-x (PMC10571431; doi:10.1186/s12913-023-10081-x)
Supplement: Supplementary file 2 — Additional file 2 [file 12913_2023_10081_MOESM2_ESM.pdf]

Mapping of leadership/management training for medical staff

Interview guide

1. What is your role in leadership/management training for medical personnel?
2. What leadership/management training for medical staff are you involved in?
3. Of these trainings, what type of audience is targeted (composition of the audience, number of people, proportion between different professions...)
4. What is the profile of the trainers (extra-medical, medical, intra/extra medical institutions, medical-extra-medical pairs)
5. What is the duration and format of the different trainings (punctual, longitudinal, etc...)
6. What is the price of the different trainings? How are they financed (employer / state / employee participation)?
7. What are the teaching methods used in your different trainings? In what proportion?
   - Classroom
   - Work in small groups
   - Role plays / simulations
   - Webinars / Online learning
   - Evaluations / personal projects
   - Other
8. How do you evaluate the learning of participants in your trainings? What feedback do they receive?
9. From the following list of skills, which ones are taught in your different trainings? Are there any others?

Knowing one's own leadership (e.g., leadership styles)		Communicating internally and externally (e.g. intra / extra institutions)	
Developing and using emotional intelligence (e.g., perceiving, using, understanding one's own or others' emotions)		Giving and receiving feedback	
Managing one's time (e.g., setting priorities / preventing burnout)		Managing a project	
Developing professionally (e.g., managing career progression)		Managing resources (e.g., developing, structuring, and managing a budget / allocating resources)	
Acting with integrity (e.g., ethically, inclusively and fairly)		Managing staff (e.g. selecting, recruiting and hiring staff / conducting appraisal interviews / setting goals / delegating tasks)	

Being a role model (e.g., inspiring others)		Managing team performance (e.g., analyzing and promoting performance)	
Managing a team (e.g. motivating a team / managing a meeting / etc.)		Managing change (e.g., implementing and supporting change / stimulating innovation)	
Being involved with team members (e.g. identifying and preventing burnout / helping with personal development / etc.)		Improving the quality/safety of care (e.g. in a unit or department)	
Building and maintaining team spirit (e.g., ensuring team cohesion)		Developing a strategic vision and long-term goals	
Managing conflicts (e.g., conflict of interest or opinion)			

10. In your opinion, what are the keys to successful leadership/management training?
11. In your opinion, what makes leadership/management training more difficult? What are the potential obstacles?
    a. What are the strengths and weaknesses of physicians with regard to leadership/management?
12. What do you think could be done to make leadership/management training more attractive? What feedback do you have from participants?
    a. Why do physicians come for training? What is their level of motivation?
    b. How can we mobilize physicians to take on the role of leader?
13. Do you have anything else on the topic of leadership/management training that you would like to discuss?
