# Supplementary material for: French-speaking Swiss physician’s perceptions and perspectives regarding their competencies and training need in leadership and management: a mixed-methods study
Source: BMC Health Serv Res. 2023 Oct 12;23:1095. doi: 10.1186/s12913-023-10081-x (PMC10571431; doi:10.1186/s12913-023-10081-x)
Supplement: Supplementary file 3 — Additional file 3 [file 12913_2023_10081_MOESM3_ESM.pdf]

<u>Information form for participants</u>

<u>Concerning</u>: "Leadership/Management Training Needs Assessment" project

Dear participant,

You are invited to participate in a study conducted by the Institute of Primary Care Medicine and the Unit of Development and Research in Medical Education under the direction of Professor N. Junod Perron.

The purpose of this study is to evaluate the training needs of redisents, chief residents, attending physicians and head of department at the HUG in the field of leadership/management. The study is part of the development of training activities considered useful for physicians in their various roles within health institutions.

The results of this research will help to improve existing leadership/management training or to develop new ones, taking into account the needs and perceptions of the participants, their reality in the field and the recommended conceptual frameworks in the field.

Your participation consists of an individual or group interview.

You are free to accept or refuse to participate in the project. You can withdraw from the project at any time without having to justify your decision.

For the purposes of the study, and subject to your consent, the interviews will be recorded. Data collected for research purposes will be coded at the time of collection. All written data that could identify you will be replaced with a code. Recorded data will be saved on an external disk that will be placed in a locked cabinet in the principal investigator's office. Audio recordings will be destroyed 2 years after the end of the study. Transcribed data will be kept for 10 years.

The analyzed results may be published in scientific journals. Confidentiality of the data will be assured, and your name or any information that could identify you will not appear anywhere. All persons involved in the study are bound by professional secrecy.

You can contact the principal investigators of the study at any time to obtain additional information:

Dr. Noëlle Junod, Unité de Développement et de Recherche en Education Médicale (UDREM) and the Institut de Médecine de Premier Recours (IMPR), HUG, Noelle.Junod@hcuge.ch.

Mr. Robin Lüchinger, PhD student, UDREM, Robin.Luechinger@unige.ch

Participant Consent Form

Concerning: "Leadership/Management Training Needs Assessment" project

If you wish to participate in this research, we thank you for completing the following consent form. We remind you that you may withdraw from the study at any time without justification, compensation or consequence. The data collected at your departure will be kept and analysed.

If after reading the document below, you wish to have more information, please do not hesitate to contact the persons mentioned as contact.

- I declare that I have been informed orally and in writing by the undersigned information officer of the objectives and progress of the project as well as the presumed effects, advantages, possible disadvantages and possible risks.
- I am participating in this study voluntarily and I accept the contents of the information sheet that has been given to me about the above-mentioned project. I have had sufficient time to make my decision.
- I have received satisfactory answers to the questions I have asked in connection with my participation in the project.
- I agree that the competent specialists of the institution, of the project leader, may consult my raw data in order to carry out controls, provided that the confidentiality of these data is strictly assured.
- I am aware that my personal data may be transmitted for research purposes within the framework of this project only and in an encrypted form.
- I can revoke my consent to participate in the study at any time without having to justify myself, without this having any negative repercussions on the continuation of my studies.

Protection des données

- The recorded data will be saved on an external disk that will be placed in a locked cabinet in the principal investigator's office.
- All persons involved in the study are bound by confidentiality.
- The data collected is coded at the time of collection. Coding means that anything that identifies you (e.g. name, address, phone number, email address etc.) is replaced by a code.
- The code remains permanently within the UDREM. If data is to be passed on to other institutions, it is always coded
- The study may be subject to on-site inspections by the authorizing ethics committee. The physician-investigator may be required to release your personal and medical data for the purpose of these inspections. The members of the ethics commission are subject to professional secrecy.
- Your identity will never appear on the Internet or in any publication.

Certification of Information Providers:

We hereby certify that we have explained to the participant the nature, importance and scope of this research. We hereby declare that we have fulfilled all obligations in connection with this research in accordance with the applicable law. Should we become aware, at any time during the course of the project, of elements that could affect the participant's consent to participate in the project, we undertake to inform him/her immediately.

Study titel:	« Leadership/management training needs assessment»
Institutions:	IMPR, HUG, Geneva<br>UDREM, Unige, Geneva
Project location:	French-speaking Switzerland - Geneva

Contact:	Dre Noëlle Junod Perron, UDREM/UNIGE et IMPR/HUG, Noelle.Junod@hcuge.ch<br><br>M. Robin Lüchinger, doctorant UDREM/UNIGE, Robin.Luechinger@unige.ch

Having read the terms of the above document, I give my consent to participate in the "Leadership/Management Training Needs Assessment" study

☐ Yes ☐ No

Last name, First name: _________________________

Location, date: __________________________
